# Global decrease in brain sodium concentration after mild traumatic brain injury

**DOI:** 10.1093/braincomms/fcab051

**Published:** 2021-03-23

**Authors:** Teresa Gerhalter, Anna M Chen, Seena Dehkharghani, Rosemary Peralta, Fatemeh Adlparvar, James S Babb, Tamara Bushnik, Jonathan M Silver, Brian S Im, Stephen P Wall, Ryan Brown, Steven H Baete, Ivan I Kirov, Guillaume Madelin

**Affiliations:** 1 Department of Radiology, Center for Biomedical Imaging, New York University Grossman School of Medicine, New York, NY 10016, USA; 2 Department of Neurology, New York University Grossman School of Medicine, New York, NY 10016, USA; 3 Department of Rehabilitation Medicine, New York University Grossman School of Medicine, New York, NY 10016, USA; 4 Department of Psychiatry, New York University Grossman School of Medicine, New York, NY 10016, USA; 5 Ronald O. Perelman Department of Emergency Medicine, New York University Grossman School of Medicine, New York, NY 10016, USA; 6 Department of Radiology, Center for Advanced Imaging Innovation and Research, New York University Grossman School of Medicine, New York, NY 10016, USA

**Keywords:** mild traumatic brain injury, sodium MRI, total sodium concentration, clinical and cognitive assessment, diffusion tensor imaging

## Abstract

The pathological cascade of tissue damage in mild traumatic brain injury is set forth by a perturbation in ionic homeostasis. However, whether this class of injury can be detected *in vivo* and serve as a surrogate marker of clinical outcome is unknown. We employ sodium MRI to test the hypotheses that regional and global total sodium concentrations: (i) are higher in patients than in controls and (ii) correlate with clinical presentation and neuropsychological function. Given the novelty of sodium imaging in traumatic brain injury, effect sizes from (i), and correlation types and strength from (ii), were compared to those obtained using standard diffusion imaging metrics. Twenty-seven patients (20 female, age 35.9 ± 12.2 years) within 2 months after injury and 19 controls were scanned with proton and sodium MRI at 3 Tesla. Total sodium concentration, fractional anisotropy and apparent diffusion coefficient were obtained with voxel averaging across 12 grey and white matter regions. Linear regression was used to obtain global grey and white matter total sodium concentrations. Patient outcome was assessed with global functioning, symptom profiles and neuropsychological function assessments. In the regional analysis, there were no statistically significant differences between patients and controls in apparent diffusion coefficient, while differences in sodium concentration and fractional anisotropy were found only in single regions. However, for each of the 12 regions, sodium concentration effect sizes were uni-directional, due to lower mean sodium concentration in patients compared to controls. Consequently, linear regression analysis found statistically significant lower global grey and white matter sodium concentrations in patients compared to controls. The strongest correlation with outcome was between global grey matter sodium concentration and the composite *z*-score from the neuropsychological testing. In conclusion, both sodium concentration and diffusion showed poor utility in differentiating patients from controls, and weak correlations with clinical presentation, when using a region-based approach. In contrast, sodium linear regression, capitalizing on partial volume correction and high sensitivity to global changes, revealed high effect sizes and associations with patient outcome. This suggests that well-recognized sodium imbalances in traumatic brain injury are (i) detectable non-invasively; (ii) non-focal; (iii) occur even when the antecedent injury is clinically mild. Finally, in contrast to our principle hypothesis, patients’ sodium concentrations were *lower* than controls, indicating that the biological effect of traumatic brain injury on the sodium homeostasis may differ from that in other neurological disorders.

Note: This figure has been annotated.

## Teaser

Gerhalter et al. report the *in vivo* detection of ionic imbalances in mild traumatic brain injury in the form of decreased total sodium concentrations in global grey and white matter compared to controls. These changes correlated with the degree of neuropsychological impairment and differentiated non-recovered patients from controls.

## Graphical Abstract

**Graphical Abstract:**
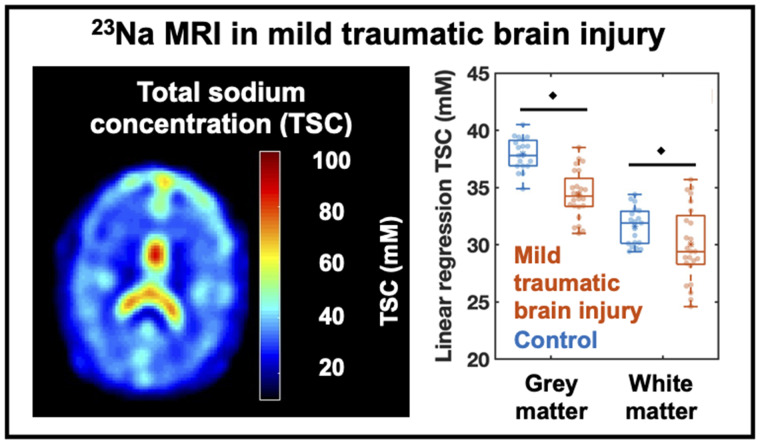

## Introduction

Traumatic brain injury (TBI) secondary to head trauma is a prevalent cause of neurological disability, with an estimated annual world incidence of 69 million.^1^ TBI survivors experience a wide range of behavioural, cognitive, emotional, and physical symptoms and disabilities. Least severe are the consequences of mild TBI (mTBI), which nevertheless accounts for the vast majority of cases, and can cause post-concussive symptoms such as headache, dizziness, fatigue, attention deficit, depression and anxiety.^2^ While most patients recover, 10–15% report persistent long-term symptoms.^3–5^ An mTBI diagnosis is based on assessments including the duration of posttraumatic amnesia, the duration of loss of consciousness and the Glasgow Coma Scale.^6^^,^^7^ The latter is a broadly used neurological assessment tool, but unfortunately, neither its score nor symptom severity correlate well with patient outcome. Clinical management and prognosis of mTBI are further hampered by the fact that conventional imaging, i.e. CT and MRI, is most often unrevealing.^8^ Therefore, alternative techniques are needed for better disease characterization and prediction of clinical outcomes.

Due to its wide availability and sensitivity to microstructural injury, diffusion tensor imaging (DTI) is at the forefront of quantitative magnetic resonance-based methods applied thus far to TBI.^9^ The main DTI metrics are the fractional anisotropy (FA), a scalar value that describes the anisotropy of water diffusion, and the apparent diffusion coefficient (ADC), a measure of the average diffusion. Unfortunately, there are often contradictory results in regard to the presence, direction, anatomical location and causative aetiology of the reported diffusional changes.^10^ Indeed, it is well-known that FA has poor specificity, since it can increase or decrease in response to a variety of pathological processes.^10^ Advanced diffusion metrics have the potential to have better utility,^11^ but the current consensus is that there is insufficient evidence for the clinical use of DTI in TBI.^12^

Much of the sensitivity of DTI is linked to the pathophysiological hallmark of TBI, diffuse axonal injury (DAI), defined as axonal varicosities resulting from cytoskeletal breakdown.^13^^,^^14^ However, the swollen axon profile significantly underestimates the total extent of injury. The TBI impact also causes molecular-level perturbations, starting with alterations in cell membrane permeability and ion transport regulation.^15^ The cell membrane, but also ion transporters, can be impaired, broken or dysregulated, leading to a cascade of metabolic processes that can include unregulated flow of extracellular sodium into the cell,^16^ provoking calcium influx and a number of downstream processes, ranging from reversible mitochondrial dysfunction to cell swelling and cell death.^17^

The purpose of this study was to investigate whether changes in ionic homeostasis after mTBI can be detected *in vivo* and have the potential to serve as a surrogate marker of clinical outcome. Sodium (^23^Na) MRI is a quantitative imaging tool that measures the total sodium concentration (TSC), a volume-weighted average of the intra- and extracellular sodium concentrations, used to detect dysregulation of sodium ion homeostasis in brain.^18^ Previous reports of utility in other neurodegenerative diseases,^19^ including those with white matter (WM) involvement, such as multiple sclerosis^20^^,^^21^ and amyotrophic lateral sclerosis,^22^ further motivated this exploratory study. Based on an expected increase in the intracellular sodium concentration in TBI, alongside a constant extracellular sodium concentration, we hypothesized that regional and global TSC will be higher in patients than in controls. In addition, since sodium imbalances reduce the capacity for generating action potentials,^23^ we hypothesized that increased TSC will correlate with increased symptomatology and neuropsychological impairment. Given the novelty of ^23^Na MRI in TBI, ^23^Na MRI effect sizes, along with correlation types and strengths, were compared to those obtained using the standard diffusion metrics of FA and ADC.

## Materials and methods

### Participants

The Institutional Review Board approved this study and written informed consent according to the Declaration of Helsinki was obtained from all participants. Patients admitted to the Emergency Department of a large urban hospital system were screened for TBI using ICD-10 codes and medical record keywords. The inclusion criteria were (i) 18–65 years of age, (ii) mTBI diagnosis according to the American Congress of Rehabilitation Medicine,^7^ (iii) less than 2 months from injury date, (iv) no blast or penetrating brain injury, (v) no previous mTBI within the past 2 years and no lifetime moderate or severe TBI and (vi) absence of disqualifying neurological and psychiatric conditions, substance abuse, and of MRI contraindications. A TBI clinic affiliated with the same hospital system served as an additional source of patients who were recruited using the same criteria. In total, 31 patients (23 female, average ± standard deviation 37.1 ± 12.4 years old, range 18–60 years) were prospectively enrolled between November 2018 and December 2019. Nineteen age- and gender-matched healthy volunteers (12 female, 31.4 ± 7.5 years old, range 23–54 years) were recruited as controls. Their inclusion criteria were identical to those of the patients, but with absence of any TBI history.

### Clinical and neuropsychological assessments

Prior to scanning, patients underwent clinical and neuropsychological assessment using tools from the National Institute of Neurological Disorders and Stroke TBI Common Data Elements (CDE) database. The CDE are consensus-based recommendations for data collection and best practices in TBI research.^24^

Global outcome and post-concussive symptomatology were assessed by the Glasgow Outcome Scale—Extended (GOSE)^25^ and the Rivermead Post-Concussion Symptoms Questionnaire (RPQ),^26^ respectively. As the only core CDE outcome measure for TBI, the eight-point GOSE is widely used and validated. It assesses the overall impact of TBI on independence, employability, cognition and social/community relationships. The RPQ is the most widely used tool to evaluate the presence and severity of post-concussive symptoms after mTBI. Patients are asked to rate their symptoms on a five-point scale from 0 (not experienced at all) to 4 (severe problem). We stratified the answers to this questionnaire according to different classification methods: (i) the RPQ total score,^26^ (ii) the RPQ 3 and RPQ 13, which groups symptoms into early and late-onset, respectively,^27^ and (iii) the three-factor model with three subscales grouping cognitive, somatic, and emotional symptoms.^28^

Neuropsychological assessment was performed with the Brief Test of Adult Cognition by Telephone (BTACT),^29^ a battery that probes five domains: episodic verbal memory, working memory, executive functioning, reasoning and speed of processing. The scoring was done as previously described.^30^ Briefly, for each individual, a *z*-score for each subtest was derived based on normative data from the MIDUS II Cognitive Project,^31^ and a composite (total) *z*-score was derived from the average of the five *z*-scores. The BTACT was performed either face-to-face prior to the MRI exam, or over the phone immediately following the in-person MRI visit. In-person and phone assessments can be used inter-changeably within a single study.^29^

### MRI acquisitions

Each participant underwent two scanning sessions on a 3 T scanner (MAGNETOM Prisma, Siemens Healthcare): ^1^H imaging with a 20-channel head coil (Siemens Healthcare) and ^23^Na imaging with a custom-engineered ^1^H/^23^Na dual-tuned birdcage head coil.

During the ^1^H session, three conventional qualitative ^1^H MRI acquisitions were performed: (i) 2D-Fluid Attenuation Inversion Recovery (repetition time/echo time/inversion time = 9000/81/2500 ms, voxel size = 0.7 × 0.7 × 5.0 mm³, acquisition time = 2:44 min), (ii) Susceptibility Weighted Imaging (repetition time/echo time = 28/20 ms, voxel size = 0.7 × 0.7 × 3.0 mm³, acquisition time = 3:46 min) and (iii) 3D-T1-weighted Magnetization Prepared Rapid Gradient Echo (high-resolution MPRAGE: repetition time/echo time/inversion time = 2400/2.24/1060 ms, voxel size = 0.8 × 0.8 × 0.8 mm^3^, acquisition time = 6:38 min). Diffusion-weighted images were acquired with a mono-polar gradient pulse echo-planar imaging (EPI) sequence (nine *b*_0_ images, six diffusion directions at *b* = 250 s/mm^2^, 60 at *b* = 1000 s/mm^2^ and 60 at *b* = 2000 s/mm^2^, repetition time/echo time = 3500/70 ms, 2 mm isotropic resolution, GRAPPA 2, multiband acceleration 2) and a strong fat suppression. Another *b*_0_ volume with opposite phase-encoding direction (i.e. posterior−anterior versus anterior−posterior) was acquired to correct EPI distortions.^32^ The total acquisition duration of the diffusion dataset was 8.8 min.

For ^23^Na MRI, low-resolution MPRAGE images (repetition time/echo time = 2100/4.2 ms, voxel size = 1.5 × 1.5 × 1.5 mm^3^, acquisition time = 3:49 min) were first acquired with the ^1^H channel for co-registration with the ^1^H MRI session. Then, a global flip angle calibration^33^ and a manual B_0_ shim using the manufacturer’s ^1^H MRI-based B_0_-shimming routine were performed. Spin-density weighted sodium images were acquired using a 3D non-Cartesian Fermat Looped Orthogonally Encoded Trajectories (FLORET) sequence^34^ with an ultrashort echo-time of 0.2 ms to reduce the loss of signal due to the fast decay of ^23^Na signal (repetition time = 100 ms, flip angle = 90°, 3 hubs at 45°, number of interleaves/hub = 26, nominal resolution = 6 × 6 × 6 mm^3^, acquisition time = 5:59 min).

### Image processing

A neuroradiologist (S.D.) with more than 10 years of experience examined all qualitative ^1^H images using the NIH CDE guidelines developed for TBI,^35^ which included haematoma, haemorrhage, diffuse or traumatic axonal injury, and brain swelling.

Post-processing for DTI was performed offline using publicly available software tools from FSL^36^ and MRTrix3.0.^37^ Diffusion datasets were corrected for thermal noise,^38^ Gibbs ringing,^39^ bias field,^40^ and motion, eddy-current, and susceptibility-induced geometric distortions.^41^ FA and ADC were calculated for each voxel.^37^

The sodium images were reconstructed offline using non-uniform fast Fourier transform^42^ and zero-filled to 1.5 mm isotropic resolution with an in-house MATLAB script. All images were visually inspected for motion artefacts. Since the small diameter of the coil prohibits the use of external references, TSC maps of the brain were computed using the vitreous humour in the eyes as internal reference (with TSC assumed to be 140 mM^43^^,^^44^) and the background noise (0 mM). Since T_1_ and T_2_ of sodium are not known for the intra- and extracellular space nor during cell swelling, we did not perform any relaxation time corrections.

### Segmentation and co-registration

**Figure 1:**
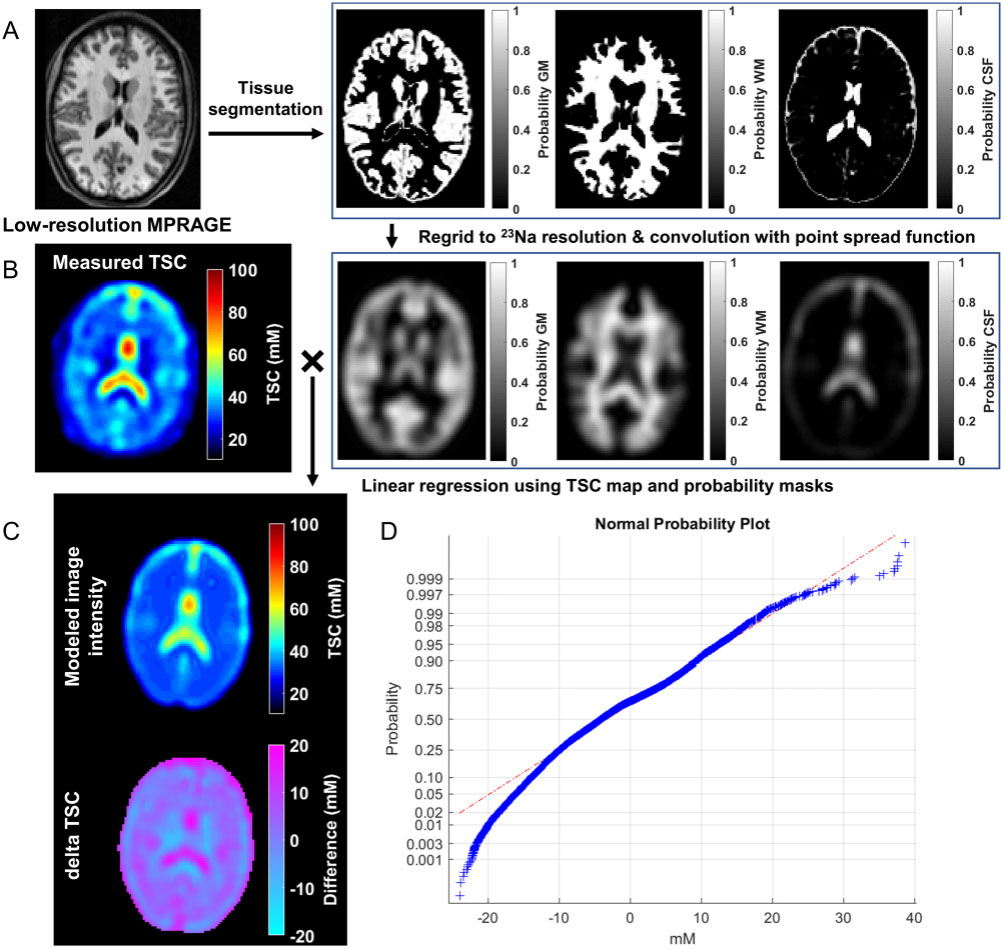
**Segmentation and linear registration for TSC.** (**A**) The low-resolution MPRAGE images with a voxel size of 1.5 mm^3^ were segmented into GM, WM and CSF masks with SPM. Our in-house software regridded these masks to the ^23^Na image resolution of 6 mm isotropic and convolved them with the point spread function. (**B**) For the whole brain tissue, we used the measured TSC map and the tissue volume fraction to solve the overdetermined equation using a linear regression approach as described in the Materials and methods section. (**C**) *Top*: the modelled image intensity distribution in the same slice as the measured TSC map, reconstructed using the global least-squares WM, GM and CSF concentrations. *Bottom*: a residual error map of the absolute value difference between measured image intensity and modelled TSC map. Note the small residual values for WM and GM demonstrating the quality of the global concentration assumption. (**D**) A normal probability plot of the error residuals from the entire brain tissue of the same subject. Most voxels lie on the red dashed line indicating a normal distribution, which points to a normal distribution of the residuals.

All masks (*n* = 12) used for obtaining voxel-averaged values of TSC, FA and ADC were extracted from the high-resolution (^1^H session) MPRAGE images (Supplementary Fig. 1). FreeSurfer version 6.0.0^45^ was used to segment the following masks: global WM, global cortical GM, and bilateral caudate, putamen, globus pallidus, and thalamus. Additional regional WM ROI were manually drawn using FireVoxel software^46^ within the body, genu, and splenium of corpus callosum (CC), bilateral corona radiata, frontal WM, and posterior WM because of their reported propensity for the development of DTI abnormalities.^35^^,^^47^ All mask outputs were visually inspected for accuracy. In order to limit the investigation to normal-appearing tissues, all MRI findings, including haemorrhages and non-specific WM abnormalities, were manually outlined on the MPRAGE image with FireVoxel, and subtracted from the ROI masks. Since cortical GM and thalamus are prone to partial volume errors from neighbouring CSF, these two ROIs were eroded by one voxel. To further account for partial volume effects, mask voxels with a CSF fraction >0.3 were excluded. Outliers, defined as values above the average plus twice the standard deviation, were also excluded for TSC, FA, and ADC calculation. The individual ROIs were registered in SPM12 (UCL, UK) using the high-resolution MPRAGE as the source image and (i) the *b*_0_ image for DTI and (ii) the low-resolution MPRAGE for sodium, as the target space. After co-registration, the ROIs were overlaid on the TSC, FA and ADC maps, as shown in Fig. 1B. Voxels within each ROI were averaged and reported as ‘ROI-based’ values.

The global GM, WM and CSF masks used for the TSC linear regression were generated using SPM12 from the low-resolution MPRAGE image (Fig. 1A). Since these images were acquired in the ^23^Na session, no co-registration was necessary.

### Linear regression for global TSC measurements

Global values of TSC in WM and GM were obtained with linear regression over all brain voxels, as commonly done with MR spectroscopic imaging.^48–50^ Linear regression increases the signal-to-noise ratio (SNR), and partial volume effects are strongly reduced. This results in high sensitivity to global changes in GM and WM.^48^ Briefly, the GM, WM and CSF probability masks were co-registered with the TSC map to yield their partial volumes in every TSC voxel. The masks were then convolved with a point spread function (full width half maximum of 2.65 pixels) to correct for the spill-over effects into GM and WM regions from CSF with high TSC (Fig. 1B). The 3D point spread function was calculated based on the FLORET sequence parameters without including the effects of T_2_ relaxation. Since the decay at 3 T of the sodium signal is not well described for each tissue, we used one point spread function for all tissue types without T_2_ correction.

The TSC signal in each voxel can be modelled as a sum of the three compartments (GM, WM and CSF), where individual concentrations C of GM and WM ($C_{\mathrm{GM}},C_{\mathrm{WM}}$) are unknown, C of CSF is known ($C_{\mathrm{CSF}}=140\;\text{mM})$ and volume fractions f are derived from the convolved probability masks:

(1) $$\mathrm{TSC}=C_{\mathrm{GM}}f_{\mathrm{GM}}+C_{\mathrm{WM}}f_{\mathrm{WM}}+C_{\mathrm{CSF}}f_{\mathrm{CSF}}.$$

All brain voxels were then used for the over-determined equations for $C_{\mathrm{GM}}$ and $C_{\mathrm{WM}}$, which were solved with least-squares optimization. Values obtained with this approach are reported as GM/WM TSC obtained from linear regression.

The absolute-value TSC residual error map obtained by subtracting the modelled TSC map (calculated from Equation (1) for each voxel using its volume fraction of GM, WM and CSF and the global least-squares concentrations from GM, WM and CSF) from the experimental map is shown in Fig. 1C. The normal probability plot for the total error for TSC over the whole brain, which is used for visual assessment of the statistical normalcy of the errors, is shown in Fig. 1D.

### Statistical analysis

The sample size was determined so that the study would have at least 80% power to detect a mean TSC difference between controls and TBI of 20%. Preliminary data had indicated that the TSC coefficient of variation (CV) was expected to be 30% among controls and 15% among patients. Based on this, the study was required to have data from at least 25 subjects per group in order to detect a 20% difference. With data from at least 25 subjects per group, the study would have at least 80% power to detect correlations of magnitude 0.316 (*R*^2^ ≥ 10%) using data from all subjects and magnitude 0.4 among TBI patients. Unfortunately, termination of research activities due to COVID-19, impeded our efforts to achieve full recruitment of control subjects. Consequently, we were unable to match controls to the three oldest patients in our sample. As described below, these patients were excluded from the comparisons with controls (but were included in the correlations with clinical outcome), yielding 24 patients and 19 controls. The decision to report results with these sample sizes was taken because TSC CVs were lower than expected, and the 20% hypothesized difference between patients and controls was largely arbitrary since there were no prior studies of ^23^Na MRI in TBI. With resulting CVs of ∼10% (for range, see Supplementary Table 1) with the new sample sizes, the study was powered to detect differences of ∼10%.

Non-parametric analyses were conducted since they are robust to violations of the assumption of normality, which is important in the present context due to the relatively small sample sizes. Patients were compared to controls without adjustment for age using an exact Mann−Whitney (MW) test. Since the association between age and TSC is unknown (i.e. may not be best modelled as linear, which would be the assumption of the age-adjustment), the three oldest non-age-matched patients were omitted from the comparisons between patients and controls. We calculated CVs (standard deviation/mean × 100) and Cohen’s *d*, which provides a measure of effect size and is defined as the difference between the concentration means divided by the pooled standard deviation:

[2] $$d=\frac{\bar{X_{p}}-\bar{X_{c}}}{\sqrt{\left[\left(n_{p}-1\right){\mathrm{SD}}_{p}^{2}+\left(n_{c}-1\right){\mathrm{SD}}_{c}^{2}\right]/\left(n_{p}+n_{p}-2\right)}}$$

where $\bar{X_{p}},\;\bar{X_{c},}\;n_{p},\;n_{c}$, and ${\mathrm{SD}}_{p},\;{\mathrm{SD}}_{c}$ denote the patients’ and controls’ mean, sample size and standard deviation, respectively.

The MW test and analysis of covariance (ANCOVA) were used to compare patients who showed recovery with patients who did not, with recovery defined by a binary GOSE outcome (=8 is recovered and <8 is non-recovered, e.g. van der Naalt et al.^51^). Comparisons were made with respect to each measure, with (ANCOVA) and without (MW) an adjustment for the elapsed time from injury, respectively. Similarly, ANCOVA and MW were used to compare recovered patients and non-recovered patients to controls. Spearman rank correlation was used to assess the association between imaging measures and BTACT and RPQ. Direct and partial correlations were computed, the latter adjusting for the elapsed time from injury to imaging. The data from all mTBI patients (including the three oldest) were used for the comparisons between the mTBI subgroups defined in terms of recovery and for all correlations. All statistical tests were conducted at the two-sided 5% significance level using SAS 9.4 software (SAS Institute, Cary, NC) and *P* values are reported without multiple comparison correction due to the exploratory nature of the study.

### Data availability

Data that support the findings in this study are available from the corresponding author upon reasonable request.

## Results

Out of the 31 patients who signed informed consent, 27 (20 female, average ± standard deviation 35.9 ± 12.2 years old, range 18–60 years) underwent the full protocol. Two patients did not complete scanning due to claustrophobia; one patient was disqualified due to presence of extensive non-specific WM disease not believed to be consistent with traumatic aetiologies, and another for inconsistent account of injury circumstances, leading to doubts as to whether the definition of mTBI was met. The demographics and characteristics of the final TBI and control cohort are compiled in Table 1. Patient scanning occurred at 22.1 ± 10.2 days from injury (range 5–53 days). The mTBI was caused by falls (30%), pedestrian-object collisions (19%), sports (15%), bicycle-pedestrian accidents (15%), bicycle falls (11%), motor vehicle accidents (7%) and assault (3%). Findings from conventional MRI are also shown in Table 1. Haemorrhagic DAI was observed in two patients, and a non-axonal shear pattern of haemorrhages was observed in one patient.

**Table 1:** Demographic and clinical characteristics of study population

			mTBI patients (*n* = 27)	Controls (*n* = 19)
Female/male			19 (70%)/8 (30%)	12 (63%)/7 (37%)
Age (years)			35.9 ± 12.4 (30)	31.4 ± 7.5 (29)
Years of education			15.9 ± 2.3 (16)	16.6 ± 3.2 (16)
MRI findings	WM hyperintensities		10 (37%)	6 (32%)
	Diffuse axonal injury		2 (7%)	0
	Haemorrhage		1 (4%)	0
Source: emergency room/clinical			12 (44%)/15 (56%)	−
Time after injury (days)			22.2 ± 10.1 (5–53)	−
Loss of consciousness		no	5 (19%)	−
		<1 min	4 (15%)	−
		1–29 min	14 (52%)	−
		unknown	4 (15%)	−
Post-traumatic amnesia		no	15 (56%)	−
		<1 min	7 (26%)	−
		1–29 min	3 (11%)	−
		1–24 h	2 (7%)	−
Alteration of consciousness		no	1 (4%)	−
		<1 min	5 (19%)	−
		1–29 min	15 (56%)	−
		30–59 min	2 (7%)	−
		1–24 h	3 (11%)	−
		1–7 days	1 (4%)	−

Median value, range or percentage of population are presented in parentheses.

### Clinical and neuropsychological assessments

All patient outcome measures are compiled in Table 2. Eight patients had recovered from their injury, as assessed by a GOSE score of 8. All but one patient presented with at least one RPQ post-concussion symptom. The most frequent symptoms were headaches (88%) and poor concentration (82%), followed by taking longer to think (63%). Patients’ median BTACT score was below that of the reference age-matched population.

**Table 2:** Clinical outcome assessments and neuropsychological data

			mTBI patients (*n* = 27)
Glasgow Outcome Scale—Extended (GOSE)	5 (lower moderate disability)		1 (4%)
	6 (upper moderate disability)		14 (52%)	
	7 (lower good recovery)		4 (15%)	
	8 (upper good recovery)		8 (30%)	
Rivermead Post-Concussion Symptoms Questionnaire (RPQ)	***RPQ total***		22 ± 11.5 (0–47)
	3-factor model	Somatic	12.3 ± 6.8 (0–30)
		Emotional	4.6 ± 4.1 (0–15)
		Cognitive	5.1 ± 3.6 (0–12)
	RPQ 3/13	RPQ 3	3.6 ± 2.5 (0–10)
		RPQ 13	19.1 ± 9.8 (0–37)
Brief Test of Adult Cognition by Telephone (BTACT)	***BTACT composite z-score***		−0.35 ± 0.67 (−1.44 to 0.86)
	Subtests *z*-score	Word list recall	−0.45 ± 1.07 (−2.67 to 2.06)
		Short-delay word list recall	−0.44 ± 0.87 (−1.75 to 1.49)
		Backward digit	−0.16 ± 0.88 (−1.39 to 1.64)
		Category fluency	0.02 ± 1.2 (−1.87 to 2.92)
		Number series	−0.63 ± 1.44 (−3.74 to 1.36)
		Backward counting	−0.5 ± 1.01 (−2.44 to 1.95)

Percentage of population or range is presented in parentheses. The Rivermead Post-Concussion Symptoms Questionnaire (RPQ) was analysed according to different classification methods: The RPQ total score,^26^ the RPQ 3 and RPQ 13,^27^ and the three-factor model.^28^ The Brief Test of Adult Cognition by Telephone (BTACT) provided sub-scores in memory, executive function, and reasoning, which are summarized in a composite *z*-score of cognitive function. Total/composite scores are in italic bold.

### ROI-based TSC and DTI

**Figure 2:**
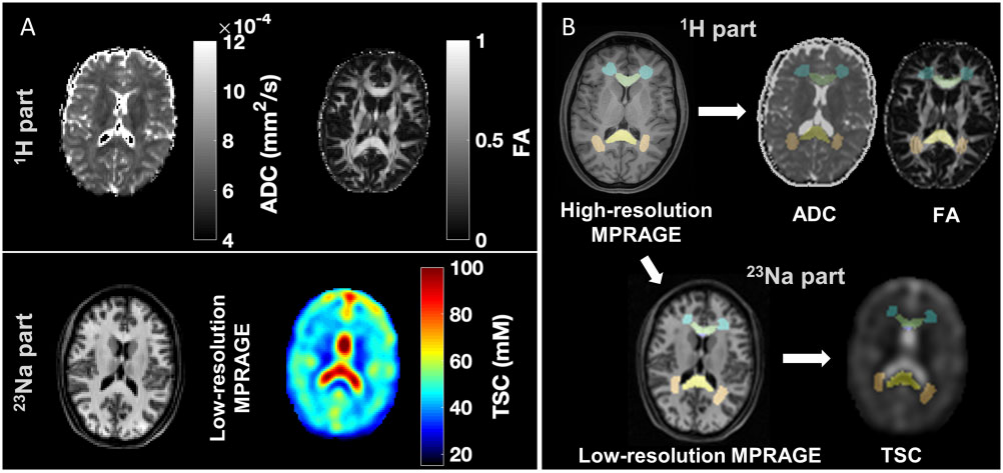
**Example of DTI and sodium MRI, segmentation and registration.** (**A**) The protocol consisted of two sessions, each done with a different RF coil. *Top*: during the ^1^H part, apparent diffusion coefficient (ADC) and fractional anisotropy (FA) maps were obtained from DTI. *Bottom*: For ^23^Na MRI, total sodium concentration maps (TSC) and low-resolution MPRAGE images for registration were acquired. (**B**) Regions of interest were obtained from high-resolution MPRAGE images and then registered to the TSC using the low-resolution MPRAGE images, and to the diffusion maps using the *b*_0_ image from the diffusion acquisition. Note the accuracy of the registration, e.g. for the genu and splenium of the corpus callosum, frontal and posterior WM (green, yellow, blue and pink, respectively).

**Figure 3:**
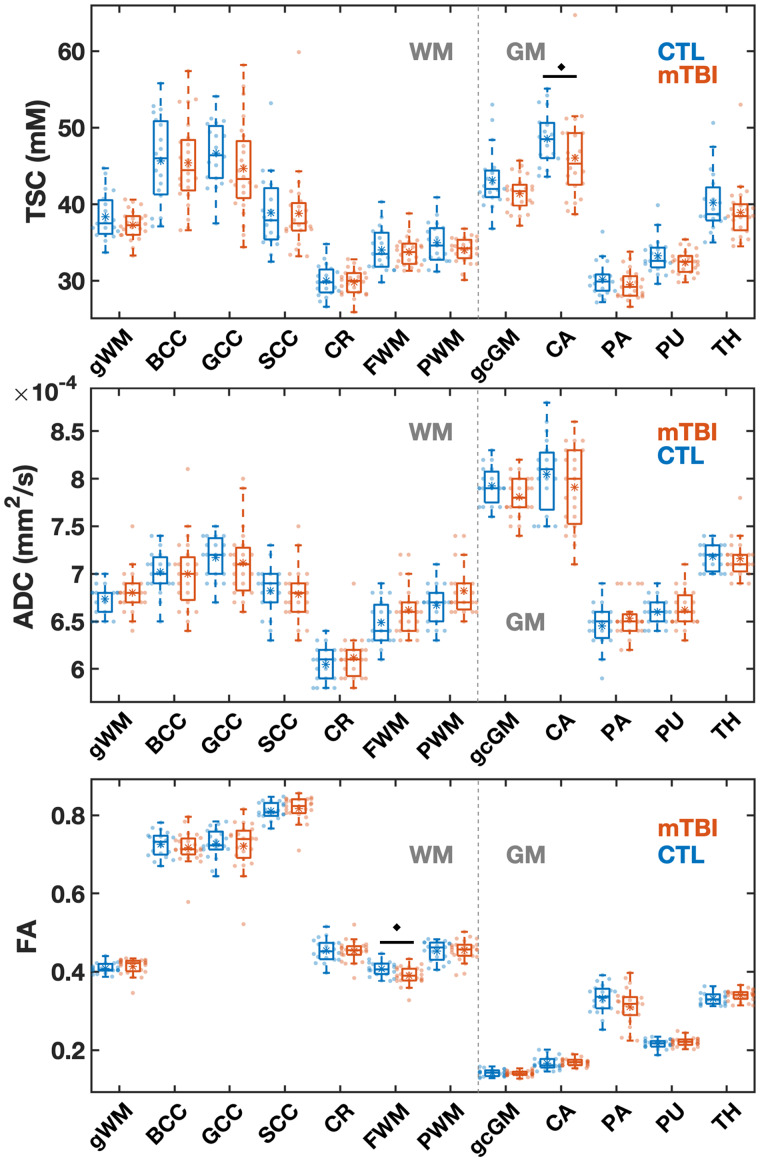
**Boxplots of TSC, ADC, and FA distributions within the mTBI and control (CTL) cohorts for the 12 ROIs.** Boxplots show the 1st, 2nd (median) and 3rd quartiles (box), ±95% (whiskers), and means (*) of the TSC, ADC and FA values obtained via voxel averaging from the global WM, global cortical GM, and regional WM and GM ROIs. The boxplots exclude the three oldest mTBI patients who lack age-matched controls. The FA in frontal white matter and the TSC in the caudate were lower in mTBI compared to controls (MW test, *filled diamonds*: *P  *<* *0.05). Note that nine out of 12 ROIs showed a lower median TSC in mTBI than in controls. gWM = global white matter; BCC = body of corpus callosum; GCC = genu of corpus callosum; SCC = splenium of corpus callosum; CR = corona radiata; FWM = frontal white matter; PWM = posterior white matter; gcGM = global cortical GM; CA = caudate; PA = pallidus; PU = putamen; TH = thalamus.

No datasets were rejected due to inaccurate segmentation or registration nor due to poor TSC or DTI quality. Example TSC, FA and ADC maps, alongside co-registered WM masks, are shown in Fig. 2. Boxplots of the TSC, ADC and FA distributions in patients and controls are presented in Fig. 3. These data in numerical form, along with effect sizes for FA and ADC, are provided in Supplementary Tables 1−3. In all ROIs, a visual trend of lower mean TSC was seen in the TBI group compared to the control group, while the only statistically significant difference was detected in the caudate (*P *=* *0.050). The Cohen’s *d* for the TSC differences between the two cohorts across all ROIs ranged from −0.02 to −0.59, with the caudate and global cortical GM showing the highest effect sizes (‘medium’ Cohen’s *d*: |0.8| > *d* > |0.5|). There were no statistically significant differences between the groups in ADC. Patient FA was normal across all regions, with the exception of lower FA in the frontal WM with a large effect size (*P *=* *0.019, Cohen’s *d* = −0.82).

### Global assessment of TSC using linear regression

**Figure 4:**
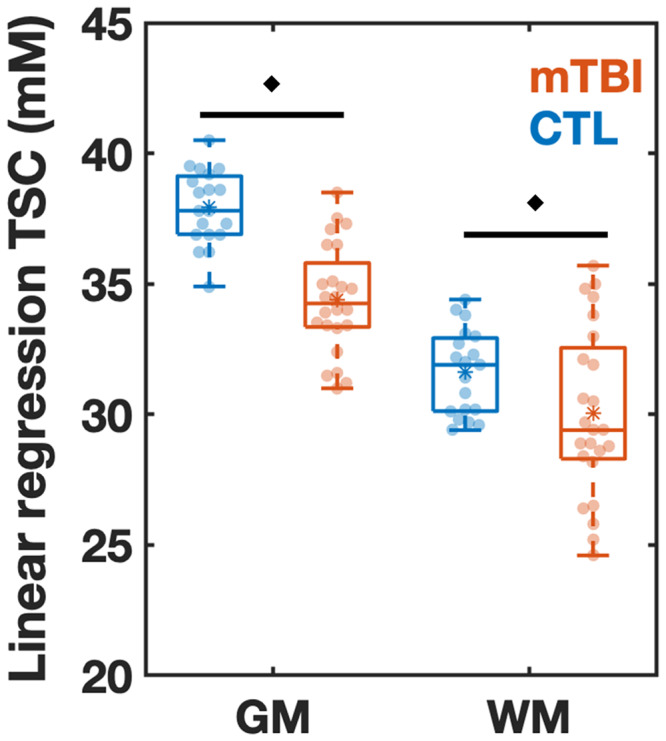
**Boxplots of TSC in mTBI and control (CTL) from linear regression analysis.** Boxplots show the 1st, 2nd (median), and 3rd quartiles (box), ±95% (whiskers), and means (*) of the TSC distributions of the controls and mTBI patients for global grey and white matter (GM, WM, respectively) using linear regression. The boxplots excluded the three elder mTBI patients who lacked an age-matched control. Note that TSC in the GM and WM was decreased in mTBI when compared to controls (MW test, *filled diamonds*: *P *<* *0.05).

Linear regresssion revealed lower GM and WM TSC in patients compared to controls, as shown in Fig. 4 and Table 3. These corresponded to a large effect size in GM (Cohen’s *d* = −1.99) and a medium effect size in WM (Cohen’s *d* = −0.61), both larger than the effect sizes of any TSC ROI-based measure.

**Table 3:** Linear regression for estimation of global grey and white matter TSC

Region	Patients (*n* = 24)					Controls (*n* = 19)					MW	Effect size
	Mean	SD	CV	Median	IQR	Mean	SD	CV	Median	IQR	*P* value	Cohen’s *d*
GM	34.4	2.1	6%	34.2	2.8	37.9	1.4	4%	37.8	2.3	**<0.001**	−**1.99**
WM	30.0	3.2	11%	29.4	4.6	31.6	1.6	5%	31.9	2.9	**0.042**	−**0.61**

Results are provided for the comparison of controls to mTBI patients after exclusion of the three oldest patients. Note the significant decrease in the grey and white matter in patients compared to controls using the exact MW test. Note that Cohen’s *d* revealed a large effect size in GM and a medium effect size in WM. Only associations in which at least one of the direct and the partial correlations was statistically significant are shown (bold, P < 0.05).

SD = standard deviation; IQR = interquartile range; GM = grey matter; WM = white matter.

### Relationships with clinical and neuropsychological assessments

Statistically significant correlations between TSC, FA and ADC and symptomatology (RPQ) and neuropsychological (BTACT) assessment are shown in Table 4. Among all ROI-based TSC data, correlations were seen only between genu TSC and the RPQ. GM and WM TSC from linear regression correlated exclusively with the BTACT, both with its composite score, and subtest scores. The strongest correlation (also across the entire dataset) was observed between GM TSC from linear regression and the composite *z*-score from the BTACT (Partial, *r *=* *0.55, *P *=* *0.005). Frontal WM FA, which was different between patients and controls, correlated with the RPQ. The FA in three other regions showed negative and positive correlations with the BTACT and RPQ, with the strongest correlation being between caudate FA and word recall subtest (Direct, *r* = −0.52, *P *=* *0.007). The ADC in two regions showed correlations with BTACT subtest scores.

**Table 4:** Correlations between MRI measures and patient outcome assessments

Imaging measure	Region	Assessment	Direct		Partial	
			*r*	*P*	*r*	*P*
ADC	Genu of CC	Number series *z*-score	0.40	**0.048**	0.44	****0.035****
	Cortical GM	Backward digit *z*-score	0.42	**0.035**	0.46	**0.027**
FA	Caudate	Emotional RPQ	−0.46	**0.018**	−0.48	**0.019**
	Caudate	Category fluency *z*-score	−0.43	**0.038**	−0.43	**0.043**
	Caudate	Word list recall *z*-score	−0.52	**0.007**	−0.50	**0.016**
	Frontal WM	RPQ 3	−0.50	**0.010**	−0.46	**0.029**
	Frontal WM	Somatic RPQ	−0.44	**0.023**	−0.45	**0.031**
	Pallidus	Cognitive RPQ	0.39	**0.048**	0.40	0.056
	Posterior WM	Short-delay word list recall *z*-score	0.49	**0.013**	0.48	**0.020**
ROI-based TSC	Genu of CC	***Total* *RPQ***	0.43	**0.027**	0.40	0.052
	Genu of CC	RPQ 13	0.44	**0.023**	0.41	**0.046**
	Genu of CC	Cognitive RPQ	0.41	**0.032**	0.46	**0.023**
Linear regression of TSC	GM	***BTACT composite z-score***	0.38	0.059	0.55	**0.005**
	GM	Short-delay word list recall *z*-score	0.40	**0.045**	0.38	0.066
	GM	Number series *z*-score	0.45	**0.020**	0.48	**0.017**
	GM	Word list recall *z*-score	0.43	**0.026**	0.47	**0.019**
	WM	***BTACT composite z-score***	0.46	**0.018**	0.41	**0.045**
	WM	Backward counting *z*-score	0.40	**0.045**	0.40	**0.050**
	WM	Number series *z*-score	0.45	**0.020**	0.48	**0.018**

Direct and partial Spearman correlations (*r*) and *P* values for the association of each imaging measure with the *z*-scores from the Brief Test of Adult Cognition by Telephone (BTACT) and Rivermead Post-Concussion Symptoms Questionnaire (RPQ) without (direct) and with (partial) adjustment for the elapsed time from injury to imaging. Total/composite scores are highlighted in italic bold. Shown are only those associations in which at least one of the direct and the partial correlations was statistically significant (bold).

CC = corpus callosum; GM = grey matter; WM = white matter; ADC = apparent diffusion coefficient; FA = fractional anisotropy; TSC = total sodium concentration.

Linear regression analysis showed lower GM TSC of both non-recovered and recovered groups compared to controls (ANCOVA *P *<* *0.001, MW *P *<* *0.001, both) and lower WM TSC in non-recovered patients compared to controls (ANCOVA *P *=* *0.034, MW *P *=* *0.038). Compared to controls, the non-recovered group had a lower FA in the frontal WM (ANCOVA *P *=* *0.012, MW *P *=* *0.008) and in the thalamus (ANCOVA *P *=* *0.048, MW *P *=* *0.041), and the recovered group had a lower ADC in the global cortical GM (ANCOVA *P *=* *0.026, MW *P *=* *0.09). The comparisons between recovered and non-recovered patients, without and with an adjustment for the elapsed time from injury showed no difference for any imaging measures within any region.

## Discussion

An important step towards effective clinical management of TBI is to understand the biological basis of post-concussive symptoms and cognitive impairment and to establish reliable, ideally non-invasive, markers for these deficits. Given the central role that ionic disequilibrium has in precipitating TBI pathophysiology,^17^ we evaluated the potential of ^23^Na MRI for providing quantitative indices related to changes in ionic homeostasis.

Due to the lack of comprehensive ^23^Na MRI studies in TBI, it was unknown (i) if abnormalities exist in different GM and WM regions; and (ii) if so, whether they would be focal, multi-focal or widespread across the entire brain. Our choice of including DTI, using regional and global ROIs and implementing two post-processing approaches (voxel averaging and linear regression) reflected our attempt to answer these questions. First, standard DTI metrics were included to serve as a ‘positive control’ in the event that sodium homeostasis proved insensitive in this setting, i.e. as evidence that brain insult sufficient to result in microstructural damage had occurred. For findings observed in both modalities, effect sizes and correlations could be compared to assess the potential clinical utility of the novel ^23^Na MRI application compared to established FA and ADC parameters. Second, as an exploratory study, ROIs were chosen to span most regions known to be susceptible to TBI, within the limited spatial resolution of ^23^Na MRI. While such approach increased the possibility of incurring type I errors, it was warranted to address both (i) and (ii), and thus to generate hypothesis testing for subsequent studies. The ROIs included cortical and deep GM to account for cognitive and somatic deficits, as well as regional and global WM for detecting the hallmark axonal injury and obtaining the benchmark WM DTI data. Third, post-processing approaches affect the SNR, and hence also the reproducibility and accuracy of the measurement. We applied our experience with ^1^H MR spectroscopic imaging (^1^H MRSI)^48^ to ^23^Na MRI, in order to study global GM and WM injury using linear regression analysis. This approach uses combined data from all brain voxels, resulting in reduced susceptibility to partial volume effects and high sensitivity to global changes. It is therefore well-suited for the study of diffuse disease,^48^ or, as in the corollary used here, to test whether an abnormality is diffusely distributed.

Our main conclusions are (i) sodium imbalances exist in mTBI and are detectable *in vivo*; (ii) they are global over the GM and WM; (iii) correlate with neuropsychological assessment; and (iv) differentiate non-recovered patients from controls. Our hypothesis on the directionality of the sodium changes was not supported, however, as lower, rather than higher, TSC was found in patients and lower TSC correlated with worse outcome.

### Diffuse TSC deficits

The above conclusions are based on the results of the linear regression approach. Its findings were especially strong for global GM TSC: among all TSC, FA and ADC comparisons, it yielded the highest effect size for differences between patients and controls. The standard, ROI-based analysis using voxel averaging, yielded statistically significant differences between the cohorts only in one ROI, but patients’ TSC distributions, means and medians were below those of controls (Fig. 3). In the context of the global linear regression results, such observation is consistent with the assertion that the lack of more ROI-based findings is due to the lower statistical power of voxel averaging versus linear regression. Indeed, the controls’ linear regression TSC CVs for GM (4%) and WM (5%) were ∼2-fold smaller than those obtained with the ROI-based approach for global cortical GM (9%) and global WM (8%). The gains in precision and sensitivity result from the use of large number of voxels and the corrections employed to control for GM/WM voxel partial volume^48^ and for ‘spill-in’ effects from CSF, whose sodium concentration is 10-fold higher than the brain parenchyma’s.

These results mirror our findings in mTBI with ^1^H MRSI. In a different cohort, we observed unidirectional effect sizes when using regional voxel averaging, without statistical significance. Linear regression, however, revealed global WM decreases of the neuronal marker *N*-acetyl-aspartate.^52^ Our results were consistent with previous ^1^H MRSI literature^53–56^ demonstrating widespread, rather than focal, metabolic injury in TBI. While the current ^23^Na MRI results are yet to be replicated, the similarities with ^1^H MRSI are noteworthy, since they suggest that compared to the macro-(anatomical MRI) and micro-(diffusional) structural phenotype of injury, physiological sequelae (metabolic and ionic) may have a more diffuse distribution. Combined ^1^H MRSI and ^23^Na MRI studies will be needed to further test this hypothesis and investigate whether any such findings share a common pathophysiological origin. Diffuse distribution of injury would also facilitate potential clinical applications because global GM/WM values would be simple to use, dispensing with the need to choose which region(s) to sample and mitigating conflicting results arising from multiple ROIs.

### Correlations with patient outcome

The main prerequisite for clinical utility is for a marker to show strong associations with relevant metrics of patient outcome. Our findings indicate that global TSC from linear regression is associated with the domains of cognitive ability and functional outcome. In the former, assessed with the BTACT, there was a directly proportional correlation with results from the neuropsychological assessment: lower GM and WM TSC were associated with worse cognitive performance, within particular subtests, as well as with the total score. GM TSC’s correlation with the total score had the highest correlation coefficient among all comparisons (cf. Table 4). The number (*n* = 7) and strength (0.3 < |*r*| < 0.5) of the TSC correlations were on the order of those obtained with FA, which were associated with sub-domains of neuropsychological function and symptomatology. Compared to GM and WM TSC, however, low FA correlated both with better or worse cognitive performance, it lacked correlations with total scores, and had fewer overall correlations with neuropsychological function. Consequently, we conclude that the GM and WM TSC results are easier to interpret and potentially less ambiguous, providing a more robust association with neuropsychological function. On the other hand, FA may be more sensitive to symptomatology: it showed two such correlations in the frontal WM, the only region which yielded a statistically significant FA difference between patients and controls.

ADC and ROI-based TSC showed far fewer associations with clinical outcome compared to TSC from linear regression and FA. Time from injury did not have a meaningful impact, with 16 out of 19 correlations showing statistical significance with and without time adjustment. The rest showed statistical significance with one test and a statistical trend with the other (0.05 < *P *<* *0.07).

In the domain of functional outcome, assessed with the GOSE, patients with incomplete recovery showed lower GM and WM TSC compared to controls, while patients with complete recovery showed lower GM, but normal WM TSC. Frontal WM FA mirrored the WM TSC findings. It is speculative to assign meaning to the different TSC findings in recovered patients, but it is noteworthy that both GM and WM TSC differentiated non-recovered patients from controls in the most commonly used clinical outcome scale in TBI.

### Possible biological basis of TSC deficit

Different underlying biological changes could cause alterations in the TSC signal. The TSC measured by ^23^Na MRI represents the volume-weighted average of intra- and extracellular sodium concentrations.^57^ Under normal conditions, the sodium concentration gradient across the membrane is 140 mM extracellular and 15 mM intracellular (with ∼80% tissue cell volume fraction), leading to a TSC of ∼40 mM.^57^ Both changes in the cell volume and in the intracellular sodium concentration therefore contribute to increases or decreases of TSC. During a TBI, the stretching of axons causes mechanical stress on membranes, altered function of sodium-potassium adenosine triphosphatase and voltage-gated sodium channels, and up-regulation of sodium channels.^17^^,^^58–60^ These mechanisms induce abnormal sodium influx and a reduced extrusion of the sodium ions out of the cell. The concomitant increase in intracellular osmolarity drives the water inflow via aquaporin channels resulting in an increase in intracellular volume fraction due to cytotoxic cell swelling.^16^^,^^61^*In vitro* experiments have shown that cell swelling occurs in both GM and WM, and that the intracellular space can increase by more than 10% after capillary occlusion.^16^ Although data on mTBI is lacking, if this process occurs, it will result in less dramatic changes in cell volume. Cell swelling of 5% and concomitant 5 mM increase in intracellular sodium would yield 38 mM TSC. However, if the sodium increase in the intracellular space is reversed by the sodium-potassium pump, but the cell remains swollen by 2%, the TSC would instead be ∼34 mM. Thus, an increase in the intracellular volume can result in decreased TSC, as observed in this study, even if the intracellular sodium concentration increases. Nevertheless, this hypothesis remains speculative in the absence of non-invasive ^23^Na MRI experiments such as inversion-recovery, multiple quantum filters, or animal experiments using of shift reagents^62–64^ to specifically detect changes in intracellular sodium concentration.

### ROI-based findings

Lastly, we discuss the ROI-based findings across all modalities independently from the linear regression results. For TSC, we consider the only prior report of ^23^Na MRI in TBI, a pilot study in 11 patients of 3 CC ROIs.^65^ Compared to controls, patients had high TSC (genu), statistical trend for low TSC (splenium) and no findings (body). In our study, these regions showed unidirectional effect sizes for lower TSC in patients, without statistical effects. A comparison between the studies, however, may not be valid due to differences in methodology and study design. Additional confounding factors include the study’s lower sample size, almost all patients having had multiple previous TBIs and some with diagnosis of epilepsy, which is known to result in higher TSC.^66^

The most frequently employed quantitative MR technique in TBI is DTI.^47^^,^^67^^,^^68^ In WM, tract anisotropy renders water diffusion highly directional, and therefore low FA and high ADC are thought to indicate damage. Indeed, we found low FA in a region known for its susceptibility to TBI, the frontal WM.^47^ There was a lack of other FA findings, and of any ADC findings, which is in line with some DTI studies in TBI, e.g. Ilvesmäki et al.,^69^ but not others, e.g. Meier et al.^70^ We note that our cohort did not have widespread abnormalities on conventional MRI, which in a large study were found to be determinants of DTI changes.^71^

### Limitations

We note several limitations. First, due to the exploratory nature of this study, we did not perform multiple comparison corrections, which increases the possibility of incurring type I errors. However, this approach is recommended for initial investigations, so that all promising results can be subject to validation studies, rather than to risk *not* reporting a true effect. Nevertheless, we note that even if corrections were applied, they would not influence the main conclusions. Specifically, with or without the statistically significant ROI-based findings (TSC in caudate, and FA in frontal WM), we can conclude that in the regional analysis ^23^Na MRI and DTI were equally (in)sensitive to mTBI. The lack of more statistically significant regional TSC findings was likely due to low statistical power, because the linear regression results indicate that TSC abnormalities are diffuse over the entire brain. Second, it remains unclear if the underlying injury is truly generalized or multifocal, i.e. whether in some ROIs the common directionality of the effect size is not due to low statistical power, but measurement noise. Indeed, some WM ROIs showed very small effect sizes, suggesting that this may be the case for some regions. In GM, the effect sizes were larger and within a narrower range, perhaps suggesting a more truly diffuse injury distribution. These questions should be addressed with analyses yielding lower regional CVs, which may be achievable with higher magnetic field strength, larger cohorts, and longer acquisition times to increase the SNR of sodium MRI in individual regions. Increased resolution would also improve regional delineation in sodium images. Third, the linear regression analysis systematically showed negative errors in areas close to the ventricles. Although the cause for this observation remains speculative, these errors might be related to signal loss in dense WM regions due to residual quadrupolar interactions as previously described by Stobbe and Beaulieu.^72^ The splenium of CC and the posterior limb of the internal capsule areas also exhibited lower TSC compared to other WM and GM areas, which would support the hypothesis of signal loss in these areas. Additional experiments would be needed to confirm this hypothesis, but are not within the scope of this study. Fourth, we did not separate intra- from extracellular sodium, which could provide more specific insights on the underlying alterations of its homeostasis. Sequences such as inversion-recovery and multiple quantum filters could be used in order to achieve a weighting towards intracellular sodium content.^73^^,^^74^ Finally, the time from injury to the MR examinations varied relatively widely (5 − 53 days) and the pathological process during this time is thought to be dynamic.^15^ This is known to affect FA, e.g. which might initially decrease and later increase, reflecting different processes.^75^ While we accounted for time from injury in our statistical analysis, such a scenario may have contributed to the lack of FA findings, and therefore future studies should compare the sensitivity of TSC with that of FA using narrower time frames. There is also a need to determine whether TSC itself is dependent on time from injury.

## Conclusion

Global GM and WM TSC obtained using linear regression revealed lower TSC in mTBI patients compared to controls. These changes correlated with the degree of neuropsychological impairment and differentiated functionally non-recovered patients from controls. Overall, the findings suggest that sodium imbalances exist in mTBI, are diffuse rather than focal, and correlate with important aspects of patient outcome. Further studies are needed to investigate the potential of ^23^Na MRI as a biomarker in TBI and to disambiguate the nature of the observed TSC decrease, which was in contrast to our hypothesis.

## Supplementary material

Supplementary material is available at *Brain Communications* online.

## Supplementary Material

fcab051_Supplementary_Data

## Abbreviations

ADC =: apparent diffusion coefficient
ANCOVA =: analysis of covariance
BTACT =: brief test of adult cognition by telephone
CC =: corpus callosum
DAI =: diffuse axonal injury
DTI =: diffusion tensor imaging
FA =: fractional anisotropy
GM =: grey matter
GOSE =: Glasgow Outcome Scale—Extended
^1^H =: proton
^1^H MRSI =: proton magnetic resonance spectroscopic imaging
MPRAGE =: Magnetization Prepared Rapid Gradient Echo
mTBI =: mild traumatic brain injury
MW =: Mann−Whitney
^23^Na =: sodium
ROI =: region-of-interest
RPQ =: Rivermead post-concussion symptoms questionnaire
SNR =: signal-to-noise ratio
TBI =: traumatic brain injury
TSC =: total sodium concentration
WM =: white matter
