# Supplementary material for: Global decrease in brain sodium concentration after mild traumatic brain injury
Source: Brain Commun. 2021 Mar 23;3(2):fcab051. doi: 10.1093/braincomms/fcab051 (PMC8066885; doi:10.1093/braincomms/fcab051)
Supplement: fcab051_Supplementary_Data [file fcab051_supplementary_data.docx]

## Supplementary material


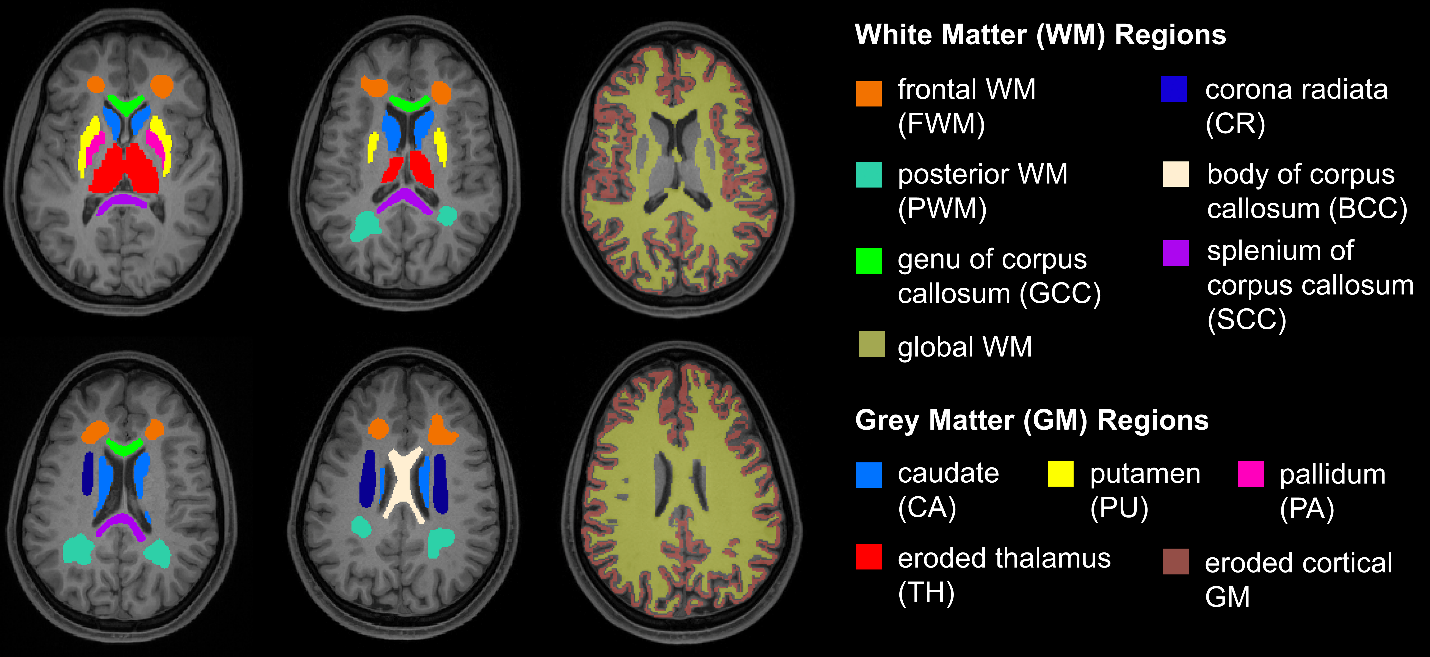


**Supplementary Figure 1**: **Regional analysis of grey and white matter.** T_1_-weighted axial MPRAGE images overlaid with the 12 ROIs in which TSC, FA, and ADC were quantified for the regional analysis. Four GM structures (thalamus, caudate, putamen, and pallidum), as well as the global WM and cortical GM, were automatically segmented using FreeSurfer, and six WM regions were manually outlined using FireVoxel (frontal WM, posterior WM, corona radiata, and body, splenium, and genu of CC). The cortical GM and thalamus masks shown have been eroded by one voxel to decrease CSF partial volume.

**Supplementary Table 1:** **The mean, standard deviation (SD), median, and interquartile range (IQR) of TSC in different regions.** Results are provided in mM for the comparison of controls to mTBI patients after exclusion of the three oldest patients. The subject groups were compared without adjustment for age using an exact Mann-Whitney (MW) test. Analysis of covariance (ANCOVA) compared the groups with adjustment for age and the effect size is measured with Cohen’s *d*. *p* values smaller than 0.05 and Cohen’s *d* values greater than |0.5| reflecting medium to large effect sizes are highlighted in bold. Abbreviations: CC: corpus callosum; FWM: frontal white matter; PWM: posterior white matter; gcGM: global cortical grey matter; gWM: global white matter.

| **TSC (mM)** | **mTBI (n = 24)** | | | | | **CTL (n = 19)** | | | | | **MW** | **Effect size** |
| --- | --- | --- | --- | --- | --- | --- | --- | --- | --- | --- | --- | --- |
| *Region* | *Mean* | *SD* | *CV* | *Median* | *IQR* | *Mean* | *SD* | *CV* | *Median* | *IQR* | *p-value* | *Cohen’s d* |
| gWM | 37.3 | 1.8 | 5% | 37.2 | 2.4 | 38.4 | 3.0 | 8% | 37.5 | 4.1 | 0.400 | -0.45 |
| CC body | 45.4 | 5.4 | 12% | 44.5 | 6.2 | 45.7 | 5.6 | 12% | 46.0 | 9.3 | 0.835 | -0.05 |
| CC genu | 44.7 | 5.9 | 13% | 43.3 | 6.8 | 46.7 | 4.0 | 9% | 46.4 | 6.5 | 0.152 | -0.39 |
| CC splenium | 38.8 | 5.3 | 14% | 37.5 | 3.4 | 38.9 | 4.8 | 12% | 37.9 | 6.2 | 0.892 | -0.02 |
| Corona radiata | 29.8 | 1.6 | 5% | 30.0 | 2.5 | 30.0 | 2.0 | 7% | 29.8 | 2.8 | 0.932 | -0.09 |
| FWM | 33.8 | 1.8 | 5% | 33.8 | 2.5 | 34.0 | 2.7 | 8% | 33.5 | 4.2 | 0.932 | -0.10 |
| PWM | 34.0 | 1.6 | 5% | 34.3 | 2.2 | 34.9 | 2.6 | 7% | 34.6 | 4.0 | 0.372 | -0.43 |
| gcGM | 41.4 | 2.2 | 5% | 41.7 | 2.5 | 43.2 | 3.8 | 9% | 42.0 | 3.3 | 0.159 | **-0.59** |
| Caudate | 46.0 | 5.5 | 12% | 45.3 | 6.7 | 48.5 | 3.5 | 7% | 48.5 | 4.5 | **0.050** | **-0.53** |
| Pallidus | 29.5 | 1.8 | 6% | 29.2 | 2.4 | 30.2 | 2.2 | 7% | 29.9 | 2.0 | 0.261 | -0.34 |
| Putamen | 32.4 | 1.5 | 5% | 32.5 | 2.0 | 33.2 | 2.3 | 7% | 32.6 | 2.3 | 0.303 | -0.44 |
| Thalamus | 38.9 | 3.6 | 9% | 38.5 | 3.3 | 40.2 | 4.0 | 10% | 38.7 | 3.8 | 0.361 | -0.34 |

**Supplementary Table 2:** **The mean, standard deviation (SD), median, and interquartile range (IQR) of ADC in different regions.** Results are provided in mm/s^2^ for the comparison of controls to mTBI patients after exclusion of the three oldest patients. The subject groups were compared without adjustment for age using an exact Mann-Whitney (MW) test. Analysis of covariance (ANCOVA) compared the groups with adjustment for age and the effect size is measured with Cohen’s *d*. *p* values smaller than 0.05 and Cohen’s *d* values greater than |0.5| reflecting medium to large effect sizes are highlighted in bold. Abbreviations: CC: corpus callosum; FWM: frontal white matter; PWM: posterior white matter; gcGM: global cortical grey matter; gWM: global white matter.

| **ADC × 10^-4^ (mm/s^2^)** | **mTBI (n = 24)** | | | | | **CTL (n = 19)** | | | | | **MW** | **Effect size** |
| --- | --- | --- | --- | --- | --- | --- | --- | --- | --- | --- | --- | --- |
| *Region* | *Mean* | *SD* | *CV* | *Median* | *IQR* | *Mean* | *SD* | *CV* | *Median* | *IQR* | *p-value* | *Cohen’s d* |
| gWM | 6.8 | 0.2 | 3% | 6.8 | 0.2 | 6.7 | 0.2 | 2% | 6.8 | 0.2 | 0.504 | 0.33 |
| CC body | 7.0 | 0.4 | 5% | 7.0 | 0.4 | 7.0 | 0.2 | 3% | 7.0 | 0.3 | 0.638 | -0.07 |
| CC genu | 7.1 | 0.3 | 5% | 7.1 | 0.4 | 7.2 | 0.2 | 3% | 7.2 | 0.4 | 0.255 | -0.19 |
| CC splenium | 6.8 | 0.3 | 4% | 6.8 | 0.3 | 6.8 | 0.3 | 4% | 6.9 | 0.3 | 0.452 | -0.13 |
| Corona radiata | 6.1 | 0.2 | 4% | 6.1 | 0.3 | 6.0 | 0.2 | 3% | 6.1 | 0.3 | 0.363 | 0.34 |
| FWM | 6.6 | 0.3 | 4% | 6.6 | 0.3 | 6.5 | 0.2 | 3% | 6.4 | 0.4 | 0.135 | **0.54** |
| PWM | 6.8 | 0.2 | 4% | 6.7 | 0.3 | 6.7 | 0.2 | 3% | 6.7 | 0.3 | 0.105 | **0.67** |
| gcGM | 7.8 | 0.2 | 3% | 7.8 | 0.3 | 7.9 | 0.2 | 2% | 7.9 | 0.3 | 0.113 | **-0.59** |
| Caudate | 7.9 | 0.4 | 5% | 8.0 | 0.8 | 8.0 | 0.4 | 5% | 8.1 | 0.5 | 0.408 | -0.34 |
| Pallidus | 6.5 | 0.2 | 3% | 6.5 | 0.1 | 6.5 | 0.2 | 3% | 6.5 | 0.3 | 0.447 | 0.38 |
| Putamen | 6.6 | 0.2 | 3% | 6.6 | 0.3 | 6.6 | 0.1 | 2% | 6.6 | 0.2 | 0.860 | 0.13 |
| Thalamus | 7.2 | 0.2 | 3% | 7.1 | 0.2 | 7.2 | 0.1 | 2% | 7.2 | 0.3 | 0.481 | -0.11 |

**Supplementary Table 3:** **The mean, standard deviation (SD), median and interquartile range (IQR) of FA in different regions.** Results are provided for the comparison of controls to mTBI patients after exclusion of the three oldest patients. The subject groups were compared without adjustment for age using an exact Mann-Whitney (MW) test. Analysis of covariance (ANCOVA) compared the groups with adjustment for age and the effect size is measured with Cohen’s *d*. *p* values smaller than 0.05 and Cohen’s *d* values greater than |0.5| reflecting medium to large effect sizes are highlighted in bold. Abbreviations: CC: corpus callosum; FWM: frontal white matter; PWM: posterior white matter; gcGM: global cortical grey matter; gWM: global white matter.

| **FA** | **mTBI (n = 24)** | | | | | **CTL (n = 19)** | | | | | **MW** | **Effect size** |
| --- | --- | --- | --- | --- | --- | --- | --- | --- | --- | --- | --- | --- |
| *Region* | *Mean* | *SD* | *CV* | *Median* | *IQR* | *Mean* | *SD* | *CV* | *Median* | *IQR* | *p-value* | *Cohen’s d* |
| gWM | 0.41 | 0.02 | 5% | 0.42 | 0.03 | 0.41 | 0.01 | 3% | 0.41 | 0.01 | 0.089 | 0.27 |
| CC body | 0.72 | 0.04 | 6% | 0.71 | 0.04 | 0.73 | 0.03 | 4% | 0.73 | 0.05 | 0.703 | -0.21 |
| CC genu | 0.72 | 0.06 | 8% | 0.74 | 0.06 | 0.73 | 0.04 | 5% | 0.72 | 0.04 | 1.00 | -0.13 |
| CC splenium | 0.82 | 0.03 | 4% | 0.82 | 0.04 | 0.81 | 0.02 | 3% | 0.81 | 0.03 | 0.151 | 0.25 |
| Corona radiata | 0.45 | 0.03 | 6% | 0.45 | 0.02 | 0.45 | 0.03 | 6% | 0.45 | 0.04 | 0.781 | -0.08 |
| FWM | 0.39 | 0.02 | 6% | 0.39 | 0.03 | 0.41 | 0.02 | 5% | 0.41 | 0.03 | **0.019** | **-0.82** |
| PWM | 0.45 | 0.03 | 6% | 0.46 | 0.03 | 0.45 | 0.03 | 6% | 0.46 | 0.04 | 0.802 | 0.10 |
| gcGM | 0.14 | 0.01 | 4% | 0.14 | 0.01 | 0.14 | 0.01 | 6% | 0.14 | 0.01 | 0.633 | -0.21 |
| Caudate | 0.17 | 0.01 | 5% | 0.17 | 0.01 | 0.17 | 0.02 | 10% | 0.16 | 0.02 | 0.282 | 0.19 |
| Pallidus | 0.31 | 0.05 | 15% | 0.32 | 0.05 | 0.33 | 0.04 | 11% | 0.34 | 0.05 | 0.128 | **-0.51** |
| Putamen | 0.22 | 0.01 | 5% | 0.22 | 0.01 | 0.22 | 0.01 | 5% | 0.22 | 0.01 | 0.318 | 0.41 |
| Thalamus | 0.34 | 0.01 | 4% | 0.34 | 0.02 | 0.33 | 0.02 | 5% | 0.33 | 0.02 | 0.061 | **0.56** |
